# Association of litter size with the ruminal microbiome structure and metabolomic profile in goats

**DOI:** 10.1038/s41598-024-66200-z

**Published:** 2024-07-05

**Authors:** Sayed Haidar Abbas Raza, Muhammad Khan, Xiaojun Ni, Xiaoqi Zhao, Hongyuan Yang, Yanting Jiang, Baiji Danzeng, Yina Ouyang, Sameer D. Pant, Ruimin Zhong, Guobo Quan

**Affiliations:** 1 Yunnan Animal Science and Veterinary Institute, Kunming City, Yunnan Province China; 2 Guangdong Provincial Key Laboratory of Food Quality and Safety/Nation-Local Joint Engineering Research Center for Machining and Safety of Livestock and Poultry Products, South China Agricultural University, Guangzhou, 510642 China; 3 Guangdong Provincial Key Laboratory of Utilization and Conservation of Food and Medicinal Resources in Northern Region, Shaoguan University, Shaoguan, 512005 China; 4 College of Animal Science and Technology, Northwest A&F University, Yangling, 712100 Shaanxi People’s Republic of China; 5 Yunnan Provincial Engineering Laboratory of Animal Genetic Resource Conservation and Germplasm Enhancement, Kunming City, Yunnan Province China; 6 Yunnan Provincial Genebank of Livestock and Poultry Genetic Resources, Kunming City, Yunnan Province China; 7 Gulbali Institute, Charles Sturt University, Wagga Wagga, NSW 2678 Australia

**Keywords:** Goat, Rumen bacteria, Rumen metabolite, Litter size, Fertility, Genetics, Molecular biology, Zoology

## Abstract

The Yunshang black goat is a renowned mutton specialist breed mainly originating from China that has excellent breeding ability with varying litter sizes. Litter size is an important factor in the economics of goat farming. However, ruminal microbiome structure might be directly or indirectly regulated by pregnancy-associated factors, including litter sizes. Therefore, the current experiment aimed to evaluate the association of different litter sizes (low versus high) with ruminal microbiome structure by 16S rRNA gene sequencing and metabolomic profiling of Yunshang black does. A total of twenty does of the Yunshang Black breed, approximately aged between 3 and 4 years, were grouped (n = 10 goats/group) into low (D-l) and high (D-h) litter groups according to their litter size (the lower group has ≤ 2 kids/litter and the high group has ≧ 3 kids/litter, respectively). All goats were sacrificed, and collected ruminal fluid samples were subjected to 16S rRNA sequencing and LC–MS/MC Analysis for ruminal microbiome and metabolomic profiling respectively. According to PCoA analysis, the ruminal microbiota was not significantly changed by the litter sizes among the groups. The *Firmicutes* and *Bacteroidetes* were the most dominant phyla, with an abundance of 55.34% and 39.62%, respectively. However, *Ruminococcaceae_UCG-009*, *Sediminispirochaeta*, and *Paraprevotella* were significantly increased in the D-h group, whereas *Ruminococcaceae_UCG-010* and *Howardella* were found to be significantly decreased in the D-l group. The metabolic profiling analysis revealed that litter size impacts metabolites as 29 and 50 metabolites in positive and negative ionic modes respectively had significant differences in their regulation. From them, 16 and 24 metabolites of the D-h group were significantly down-regulated in the positive ionic mode, while 26 metabolites were up-regulated in the negative ionic mode for the same group. The most vibrant identified metabolites, including methyl linoleate, acetylursolic acid, O-desmethyl venlafaxine glucuronide, melanostatin, and arginyl-hydroxyproline, are involved in multiple biochemical processes relevant to rumen roles. The identified differential metabolites were significantly enriched in 12 different pathways including protein digestion and absorption, glycerophospholipid metabolism, regulation of lipolysis in adipocytes, and the mTOR signaling pathway. Spearman’s correlation coefficient analysis indicated that metabolites and microbial communities were tightly correlated and had significant differences between the D-l and D-h groups. Based on the results, the present study provides novel insights into the regulation mechanisms of the rumen microbiota and metabolomic profiles leading to different fertility in goats, which can give breeders some enlightenments to further improve the fertility of Yunshang Black goats.

## Introduction

*Capra hircus* (Goat) is the oldest domesticated socio-economical small ruminant animal; having more than 1000 breeds across the world, they use low-quality roughage-based diets and produce a range of valuable products including meat, milk, fiber, and hides for human consumption. Moreover, owning sophisticated inherited traits such as excellent breeding efficiency makes them for poverty elevation and better return over investment. Litter size is the most essential trait in goat farming because of its direct influence on farming profitability. Recently, researchers using them as an ideal model to investigate some potential genetic as well as molecular markers associated with important traits like growth, reproduction, milk, and wool to enhance the cost-effective genomic pool^1^. Yunshang Black goat is a meat breed goats that was developed in Yunnan province, China. This breed has faster growth rates with excellent breeding efficiency as compared to other native breeds^2^. Moreover, the meat quality is remarkable and comparable with international breeds^3,4^.

The rumen of goats hosts trillions of diversified microbial populations that play a significant role in metabolic pathways and ultimately these animals’ production as well as reproduction performance^5^. The main cohort of these microbes is based upon bacterial papulation that ferments the dietary parts such as fiber (cellulolytic, and hemicellulolytic species), starch (amylolytic species), fat (lipolytic species), and protein (proteolytic species), and microbial protein synthesis^6^ to meet the host bioenergetics, and protein requirements respectively^7^. The protein could be a primary limiting factor that influences ruminal microbial characteristics as it is established that it is utilized to synthesize microbial protein (MP) utilized by the host’s *Bacteroidetes* and *Prevotella* phyla^8,9^. Therefore, protein utilization is closely linked to the composition and diversity of gastrointestinal bacterial communities that in turn significantly influence the health status and productivity of the host animal^10,11^. Recently microbial insights revealed that maternal gut and reproductive tract microbiota influence fetal microbiota composition and health^12,13^. The functioning of the rumen microbiota contributes significantly to the adaptability of ruminants to diverse diets^14^. The dietary modulation of sows’ microbiome during pregnancy improved the reproductive performance^15^.

It is established that litter size influences kids’ growth rates and survivability. A study in the recent past was carried out by Wang et al.^5^ to investigate the rumen microbiota and ruminal fermentation efficiency of six-month-old goats selected from single, twin, and triplet litter sizes, respectively. The rumen fermentation analysis revealed that the goats produced in a triplet litter have relatively lower volatile fatty acids (VFA), unsaturated fatty acids, branch chains, and essential amino acids production that led to lower body weight gain, and average daily gain. Moreover, their results showed that litter size has also a significant impact on ruminal microbiota as they found a lower ratio of *Firmicutes* and *Bacteroidota* in goats produced in triplet litter size compared to goats produced in either single or duplet litter sizes. All the above research was carried out in animals originating from different litter sizes. However, data integrating the information regarding the association between different litter sizes and how it can impact the rumen microbiome and metabolomics of the dam is unclear.

Therefore, the overall objective of this study was to characterize ruminal microbiota and metabolite profiles of the dams of Yunshang black goats having low or high litter sizes, via 16S rRNA sequencing and LC–MS/MC analysis.

## Materials and methods

### Ethics statement and approval

All experiment procedures of the current study were approved by the ethics committee of Yunnan Animal Science and Veterinary Institute (Approval No. 202009005). Further, the experiment was in line with the guidelines of the State Science and Technology Commission of the People’s Republic of China (1988), and the Standing Committee of Yunnan Provincial People’s Congress (2007.10).

### Experimental station, layout, and ruminal fluid collection

This experiment was conducted at Kunming Yixingheng Husbandry Technology Co., Ltd, located in Kunming City, People’s Republic of China (26°22′ N; 103°40′ E). A total of twenty goats of the Yunshang black breed approximately aged between 3 and 4 years were selected by following their minimum two years previous reproduction records and grouped (n = 10 goats/group) into low (D-l) and High (D-h) litter groups according to their litter size (the lower group has ≤ 2 kids/litter and the high group has ≧ 3 kids/litter, respectively). The goats were housed in their respective group with the allocation of 1 m^2^/goat-covered area in the shed. The temperature of the shed was maintained at approximately 20 °C. Both groups were fed twice daily at 8:00 and 16:00 h of the day on the same diet (detailed feed formulation along with compositional characteristics are given in Table 1). The goats were given free choice access to fresh and clean water to ensure the ad libitum intake. At the end of the experiment, the goats were fasted for 12 h before sacrificing, ruminal fluid of each goat was collected within 10 min after scarification by using a 50 mL sterile syringe, and approximately 10 mL of rumen fluid was collected into 15 mL labeled sterile freezing tube (BIOFIL, China). The collected samples were immediately frozen in liquid nitrogen, transported to the laboratory by using a liquid nitrogen container and stored at − 80 °C for the analysis.

**Table 1:** Feed formulation and nutrient composition of the experimental diet on a dry basis.

Item	Inclusion level (%)
Corn grains ground	28.00
Soybean meal (48%)	20.00
Broad bean stem and leaf bran	10.00
Alfalfa granule	18.00
Corn silage	20.00
CaHPO_4_	1.50
Premix^1)^	1.00
NaCl	0.50
NaHCO_3_	1.00
Nutrient	Concentration
DE/(MJ/kg)^2)^	13.26
Crude protien (%)	16.35
Neutral detergent fiber (%)	22.36
Acid detergent fiber (%)	21.29
Calcium (%)	0.90
Phosphorous (%)	0.55

^1^The premix provided the following per kg of diets: Mn 98 mg, Fe 245 mg, Zn 80 mg, Cu 10 mg, I 2.5 mg, Se 0.65 mg, Co 0.65 mg, VA 10,000 IU, VD_3_ 1000 IU, VE 50 IU.

^2^The digestibility of concentrates is calculated using the data in the “Mutton Sheep Feeding Standards NY/T 816-2004”; the digestibility of coarse feeds is calculated according to the following formula: DE = 16.96–0.165NDF + 0.212CP.

### Microbial DNA extraction

The collected rumen fluid samples were thawed, filtered by using four-layer sterile gauze, and centrifuged at 12,000*g* for 10 min by maintaining 4 °C temperature of the centrifuge machine. The sedimental part was collected and DNA extraction was carried out by using commercial kits (E.Z.N.A. ®Stool DNA Kit D4015, Omega, Inc., USA) according to the given instructions of the manufacturer company. During extraction, nuclear-free water was run as the blank. The total DNA was eluted using 50 mL of the Elution buffer and finally stored at − 80 °C.

### PCR amplification and 16S rRNA sequencing

The V3-V4 region of the prokaryotic (bacterial and archaeal) small-subunit (16S) rRNA gene was amplified using primers 341F (5’-CCTACGGGNGGCWGCAG-3’) and 805R (5’-GACTACHVG GGTATCTAATCC-3’). The 5’ ends of the primers were tagged with specific barcodes per sample and sequenced using universal primers. The PCR amplification was performed in a total volume of 25 mL reaction mixture containing 25 ng of template DNA, 12.5 mL PCR premix, 2.5 mL of each primer, and PCR-grade water to adjust the total volume. The PCR settings to amplify the prokaryotic 16S fragments consisted of an initial denaturation at 98 °C for 30 s, 32 cycles of denaturation at 98 °C for 10 s, annealing at 54 °C for 30 s, extension at 72 °C for 45 s, and then final extension at 72 °C for 10 min. The PCR products were further evaluated using 2% agarose gel electrophoresis. Throughout the DNA extraction process, ultrapure water was used as the negative control to exclude the possibility of false-positive results. The PCR products were purified using AMPure XT beads (Beckman Coulter Genomics, Danvers, MA, USA) and quantified using a Qubit (Invitrogen, USA). Amplicon pools were prepared for sequencing. The size and quantity of the amplicon library were assessed using an Agilent 2100 Bioanalyzer (Agilent, USA) and Library Quantification Kit for Illumina (Kapa Biosciences, Woburn, MA, USA), respectively. Libraries were sequenced on a NovaSeq PE250 platform.

### Bacterial bioinformatic analysis

Paired-end reads were assigned to respective samples based on their unique barcode and subsequently truncated by removing the barcode and primer sequence. Paired-end reads were merged using FLASH (v1.2.8) (for 16S), and quality filtered using fqtrim (v0.94). Chimeric sequences were filtered using Vsearch software (v2.3.4), and dereplication was performed using DADA2, which then yielded feature tables and feature sequences. Alpha and beta diversity indices were subsequently calculated by QIIME2, by randomly extracting the same number of sequences (equivalent to the minimum number of sequences available for all samples) from all samples. The relative abundance (microbial counts/total counts) was also computed at different taxonomical levels. Plots involving alpha and beta diversity indices computed by QIIME2 were generated using R (v3.5.2). For species annotation, sequence alignment was performed via BLAST, utilizing SILVA and NT-16S as the alignment databases.

### Metabolite extraction and LC–MS/MC analysis

All 20 rumen fluid samples underwent sample preparation and metabolite extraction for subsequent LC–MS/MS analysis based on protocols provided by Hangzhou Lianchuan Biotechnology Co., Ltd. Briefly, stored ruminal fluid samples were thawed on ice, 20 μL sample and 120 μL of 50% pre-cooled methanol (− 20 °C) were mixed, vortexed for 1 min, and incubated at room temperature for 10 min. The extracted samples were then stored overnight at − 20 °C. After centrifugation at 4000*g* for 20 min, the supernatants were collected and transferred into a new 96‐well plate. Finally, the samples were stored at − 80 °C before the LC‐MS analysis. In addition, the pooled QC samples were also prepared by combining 10 μL of each extracted sample. The plates were immediately stored at − 80 °C before LC–MS.

Chromatographic separations were conducted using an ultra-performance liquid chromatography (UPLC) system (SCIEX, Macclesfield, UK), with reversed-phase separation on an ACQUITY UPLC T3 column (100 mm × 2.1 mm, 1.8 μm, Waters, Wilmslow, UK), at a 35 °C column temperature and a 0.4 mL/min flow rate. The mobile phase comprised solvent A (water, 0.1% formic acid) and solvent B (phenol, 0.1% formic acid). The gradient elution conditions were 0–0.5 min, 5% B; 0.5–7 min, 5% to 100% B; 7–8 min, 100% B; 8–8.1 min, 100% to 5% B; 8.1–10 min, 5% B. The injection volume for each sample was 4 μL.

Eluted metabolites were detected using a TripleTOF5600plus (SCIEX, Macclesfield, UK) mass spectrometer, configured to operate in positive and negative ionic modes. The QTOF parameters included a curtain gas set to 30 PSI, ion source gas1 and gas2 set to 60 PSI, and an interface heater temperature of 650 °C. The ion spray voltage floating was set to 5000 V and 4500 V for positive and negative ionic modes, respectively. Data collection utilized the IDA mode, with TOF’s mass range adjusted from 60 to 1200 Da. Survey scans were acquired in 150 ms with up to 12 product ion scans, and the total cycle time was set at 0.56 s. A 40 GHz multichannel TDC detector with four anode/channel detectors was monitored by four-time bins summation per scan, adjusting the pulse frequency at 11 kHz. The dynamic exclusion timer was set to 4 s. Mass accuracy was calibrated after every 20 samples during acquisition. Additionally, a quality control sample (pool of all samples) was acquired after every 10 samples to assess LC–MS stability throughout the acquisition process.

### Bioinformatic analysis of metabolome

The peak picking, peak grouping, retention time correction, second peak grouping, and isotope and adduct annotation of the MS data file were processed using the XCMS software. The raw data containing the LC–MS files were converted to mzXML format using the R packages including XCMS (Online, version 3.4.4), CAMERA (version 1.38.1), and MetaX (version 2.29). The ionic identification was carried out by combining the retention time (RT) and m/z values. The three-dimensional matrix was generated with the arbitrarily assigned peak indices (retention time m/z value of pairs), sample names (observations), and ion intensity information (variables). The metabolites were annotated using the online KEGG (http://www.genome.jp/kegg/, 29 July 2023) and HMDB databases (https://hmdb.ca/, 29 July 2023) by matching the exact molecular mass data (m/z) of the samples with those from the database^16^. The metabolites were annotated by adjusting the mass difference between the observed and database values < 10 ppm, their molecular formula was identified, and they were validated using the isotopic distribution measurements. An in-house fragment spectrum library was also used to validate the metabolite identification. MetaX was used to preprocess the peak data’s intensity further. Those features found in less than 50% of QC samples or 80% of biological samples were removed, and the remaining peaks with missing values were imputed using the k-nearest-neighbor algorithm to improve the data quality. The log-transformed (base 10) data were scaled using the central mean divided by the standard deviation of the variables. The normalized data were subjected to principal component analysis (PCA) and orthogonal projection to latent structures discriminant analysis (OPLS-DA) to compare the differences among different metabolites using Metabo-Analyst 5.0 (https://www.metaboanalyst.ca/, 29 July 2023). Metabolite screening was carried out by variable importance in projection (VIP) scores of the OPLS-DA model and statistical significance as per Student’s t-tests. Using the preprocessed dataset, PCA was used to detect outliers and evaluate batch effects. To minimize signal intensity drift over time, a robust LOESS signal correction based on quality control was fitted to the QC data concerning the order of injection. Furthermore, the relative standard deviations of the metabolic features were calculated across all QC samples, and those greater than 30% were eliminated.

### Ethics approval and consent to participate

All experiment trials conducted as part of the current study were approved by the ethics committee of the Yunnan Animal Science and Veterinary Institute (Approval No. 202009005). All investigators involved in the experimental trials strictly followed the protocols and guidelines approved by the State Science and Technology Commission of the People’s Republic of China (1988), and the Standing Committee of Yunnan Provincial People’s Congress (2007.10). Furthermore, all applicable rules and regulations of the organization and government were followed concerning the ethical use of experimental animals., as well as with the endorsement of the Ethics Committee and ARRIVE guidelines.

## Results

### Analysis of variation in metabolite

**Figure 1:**
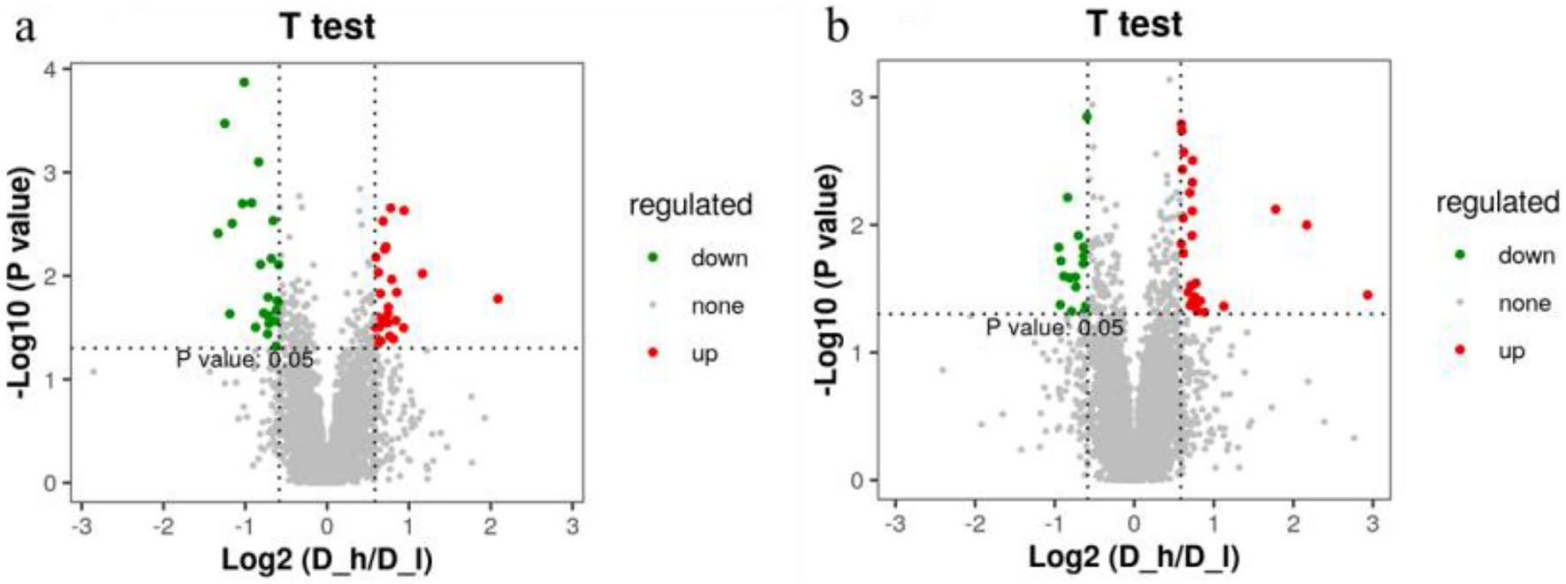
Volcano plot of metabolites. Each point represents a metabolite. Significant up-regulated metabolites are indicated in red, significantly down-regulated metabolites are indicated in green, and non-significant differences in metabolites are in grey. (**a**) Negative ion mode. (**b**) Positive ion mode.

The Variable Importance in Projection (VIP) analysis was performed to identify metabolites contributing to the variability in the metabolomic profiles of the high and low litter size groups. Based on a VIP exceeding 2 within 95% jack-knifed confidence intervals, a total of 95 differentially abundant metabolites were identified between the D-h and D-l groups for both positive and negative ionic modes (Fig. 1a,b). In negative ionic mode, 29 metabolites were significantly up-regulated while 16 were down-regulated for the D-h group (Fig. 1a). In positive ionic mode, the 28 and 22 metabolites had up and down regulation respectively for the D-h group (Fig. 1b). The metabolites that had distinguished abundance among groups were methyl linoleate, acetylursolic acid, O-desmethyl venlafaxine glucuronide, melanostatin, and arginyl-hydroxyproline. These all are known to be involved in diverse biochemical processes in the gut.

**Figure 2:**
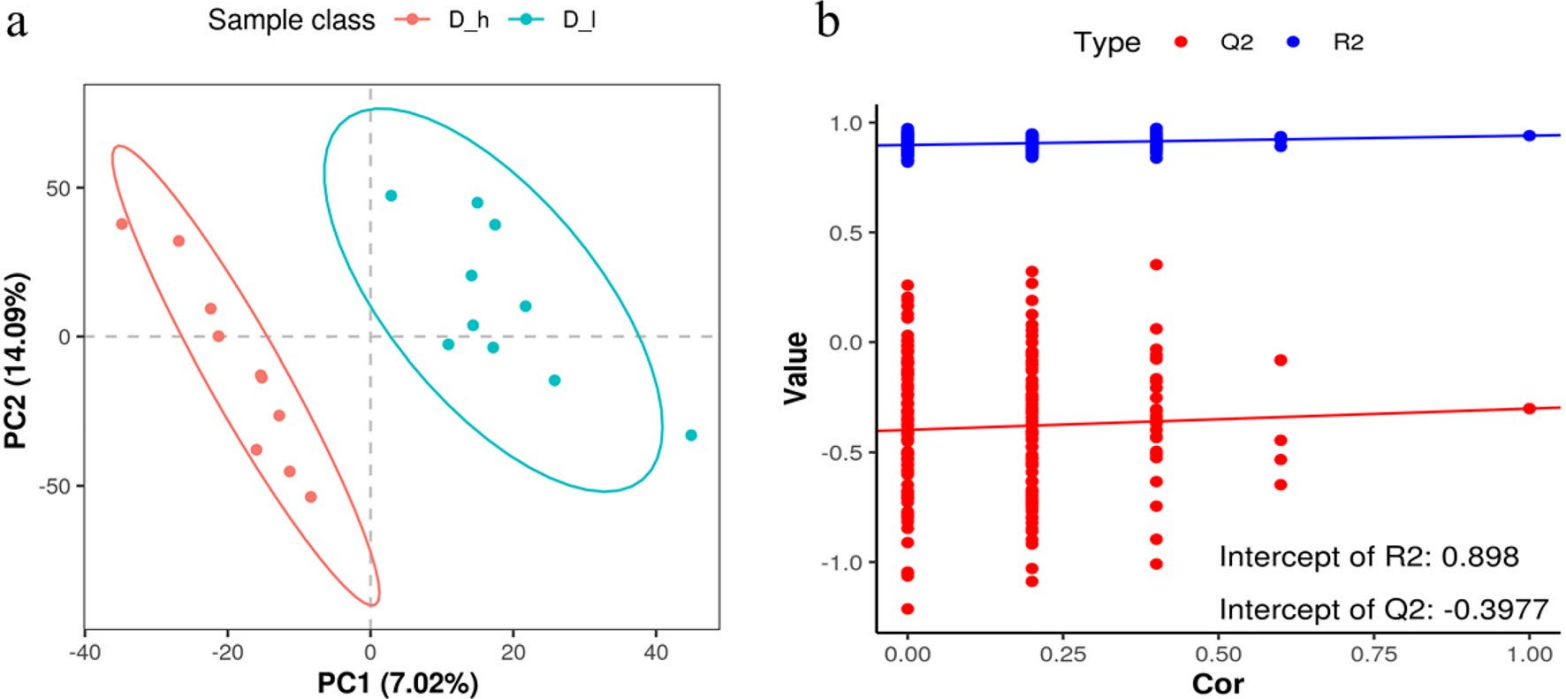
PLS-DA analysis (**a**) and Permutation test diagram (**b**).

**Figure 3:**
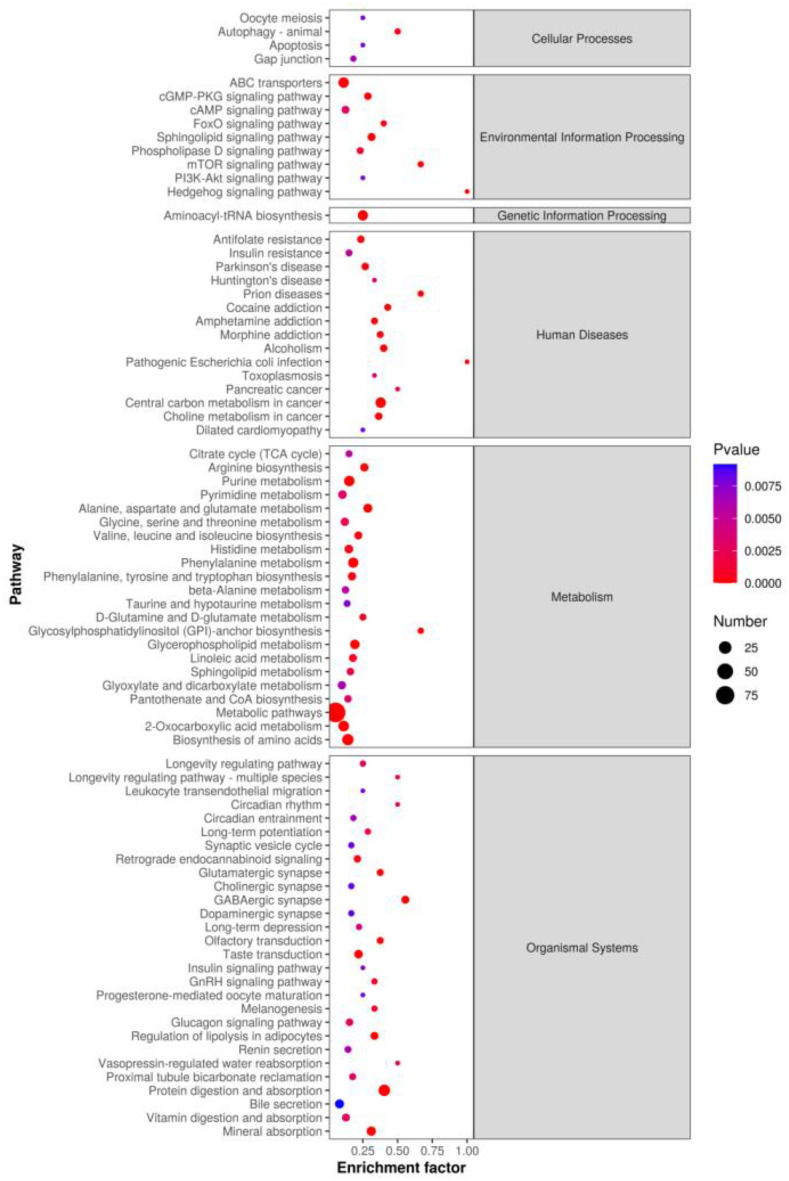
Enriched Kyoto Encyclopedia of Genes and Genomes (KEGG) pathways of the comparison between the S–h and S-l groups (only those with a *P* < 0.05 are shown).

In contrast to Principal Component Analysis (PCA) which is unsupervised, Partial Least Squares Discriminant Analysis (PLS-DA) offers a supervised method to discriminate and identify variables that maximally explain the difference between the low and high litter size groups. Figure 2a illustrates a significant separation between the D_h and D_l group based on a q-value of < 0.05 and VIP of > 1.0. The values for R^2^X and Q^2^, coupled with the outcomes of permutation tests, collectively indicate that sample quality was reasonable, as demonstrated in Fig. 2b. To identify key biochemical pathways that involved the differentially abundant metabolites, pathway enrichment analysis was conducted using the Kyoto Encyclopedia of Genes and Genomes (KEGG). Differentially abundant metabolites were found to enrich 80 pathways, 12 of which were significantly enriched, including protein digestion and absorption, glycerophospholipid metabolism, regulation of lipolysis in adipocytes, and mTOR signaling pathway (*P* < 0.05, Fig. 3).

### Analysis of variation in microbial communities

**Figure 4:**
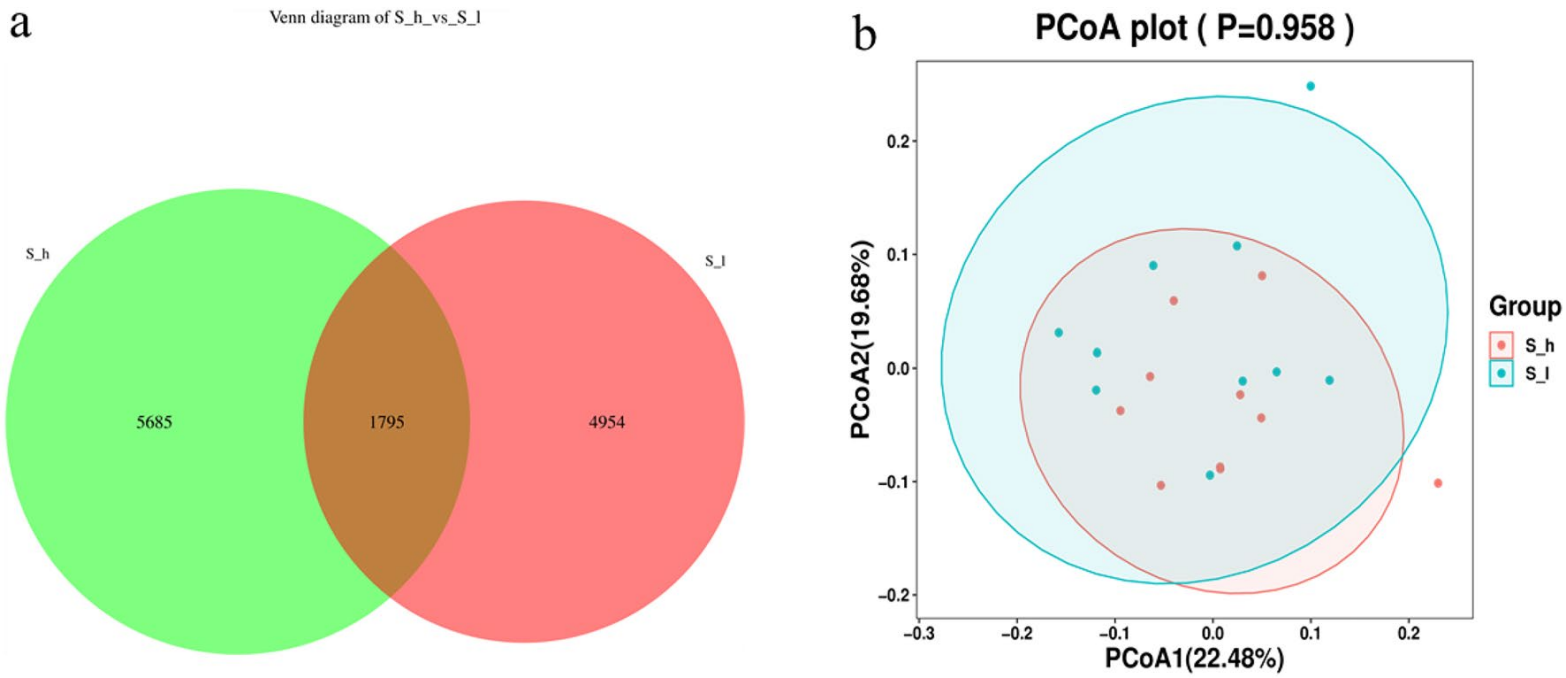
The alpha diversity of bacteria community and OTUs. (**a**) Venn diagram test of bacteria. (**b**) PCoA analysis. Red dots indicate S_h samples, green diamonds indicate S_l and green/red ellipses represent 95% confidence regions.

A total of 1,607,339 raw reads were generated upon sequencing, with sequence reads per sample ranging from 60,613 to 87,427. Subsequent analysis identified a total of 12,434 observed Operational Taxonomic Units (OTUs) based on a sequence identification of ≥ 97% between reads, and the observed OTUs were significantly higher in the D_h group. A total of 5685 OTUs were found to be unique to the D_h group, while 4954 OTUs were found to be unique to the D_l group (Fig. 4a). Principal coordinate analysis based on binary-Jaccard distances indicated that the first two axes explained 22.48% and 19.68% of overall variability, but did not indicate an obvious separation or clustering of the two treatment groups (Fig. 4b). Alpha diversity analyses based on Shannon and Simpson indices did not indicate significant differences between the D h and D_l treatment groups (*P* > 0.05).

**Figure 5:**
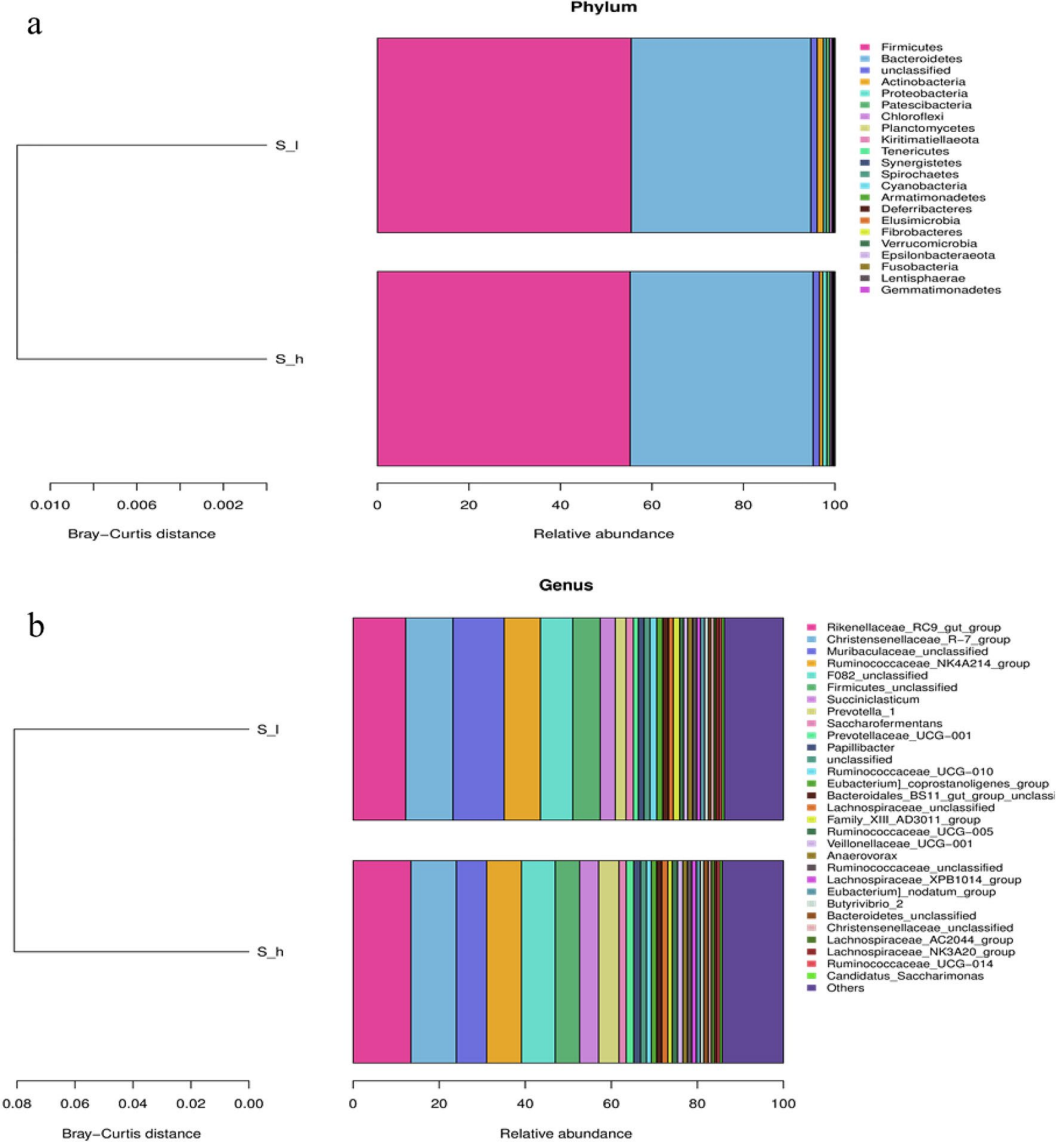
The relative abundance of bacteria in the rumen of goats at the phylum level and genus level. (**a**) Differential bacteria in the rumen at the phylum level. (**b**) Differential bacteria in the rumen at the genus level.

Furthermore, results indicated that *Firmicutes* and *Bacteroidetes* were the dominant phyla in the rumen, accounting for 55.34% and 39.62% of all phyla, respectively. A significantly higher relative abundance of *Proteobacteria* (*P* < 0.001) was observed in the D_h group (Fig. 5a). At the genus level, *Rikenellaceae_RC9_gut_group* and *Christensenellaceae_R-7_group* were the dominant genera, accounting for 12.82% and 10.77% of the relative abundance, respectively. The *Ruminococcaceae_UCG-010*, *Sediminispirochaeta*, and *Paraprevotella* were significantly more abundant in the D_h group (*P* < 0.05); however, *Howardella* was significantly less abundant in the D_h group (*P* < 0.05) (Fig. 5b).

### Correlation analysis between metabolome and microbiome

**Figure 6:**
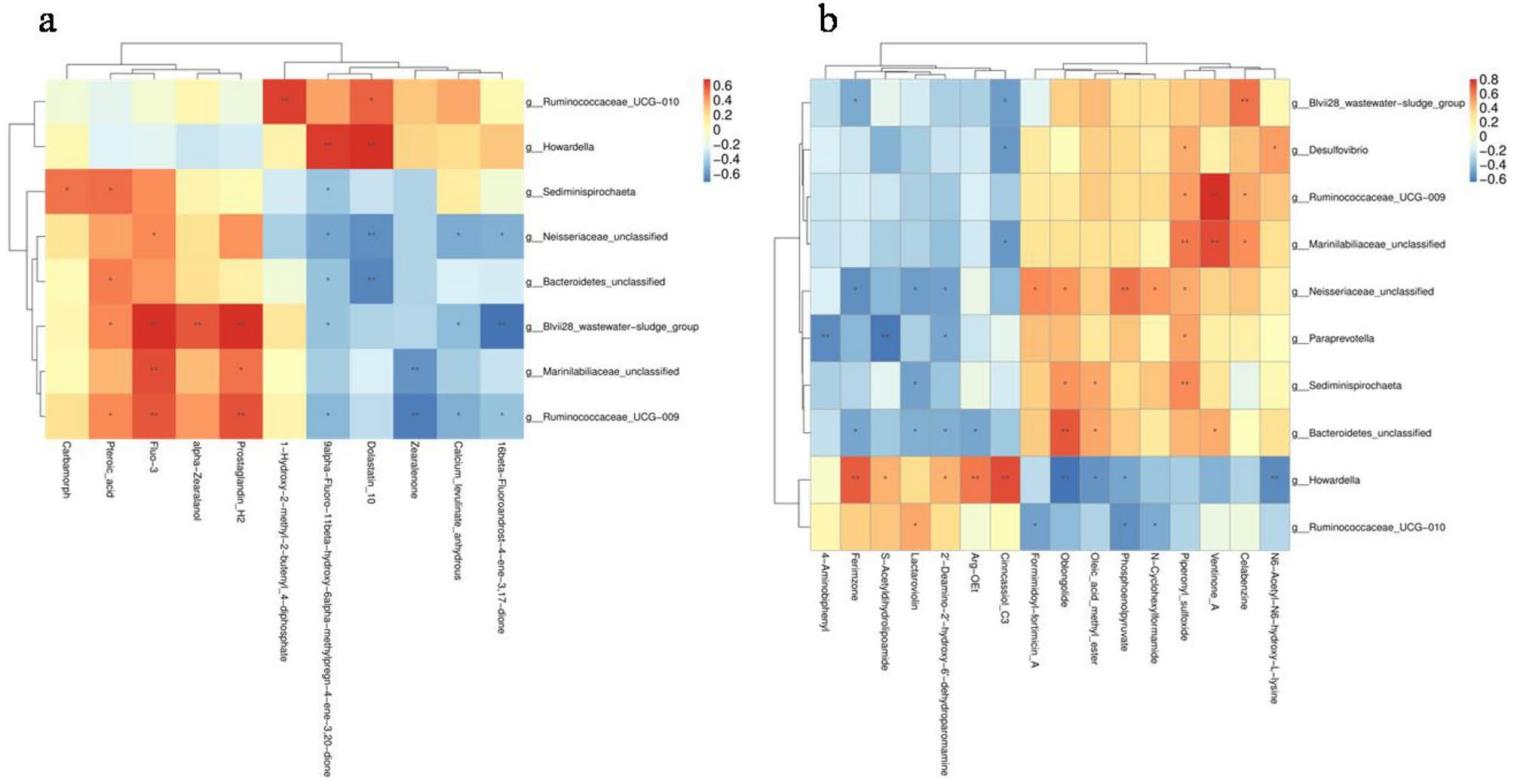
Heatmap of Spearman’s rank correlation coefficients of the relative abundances of different microbial communities and metabolites. (**a**) Negative ion mode. (**b**) Positive ion mode. Each square represents a Pearson correlation coefficient, while the gradation of color represents the size of each correlation coefficient. The red color represents a positive correlation, while the blue color represents a negative correlation. *P < 0.05, **P < 0.01.

The correlation matrix was computed using Spearman’s correlation coefficient to investigate the functional correlations between metabolites and microbial communities. Results revealed significant differences between the high and low litter size groups. In the negative ion mode, *Ruminococcaceae_UCG-010* had a significant positive correlation with the 1-Hydroxy-2-methyl-2-butenyl_4-diphosphate. Similarly, *Howardella* had a significant positive correlation with 9alpha-Fluoro-11beta-hydroxy-6alpha-methylpregn-4-ene-3,20-dione and Dolastatin_10. Additionally, *Ruminococcaceae_UCG-009* was found to be positively and significantly correlated with Pteroic_acid, Fluo-3, and Prostaglandin_H2; and negatively and significantly associated with 9alpha-Fluoro-11beta-hydroxy-6alpha-methylpregn-4-ene-3,20-dione, Zearalenone, Calcium_levulinate_anhydrous and 16beta-Fluoroandrost-4-ene-3,17-dione (Fig. 6a). In the positive ion mode, *Ruminococcaceae_UCG-009* and *Marinilabiliaceae_unclassified* were positively and significantly correlated with Piperonyl_sulfoxide, Ventinone_A, and Celabenzine, respectively. The *Howardella* were negatively and significantly correlated with Oblongolide, Oleic_acid_methyl_ester, Phosphoenolpyruvate, and N6-Acetyl-N6-hydroxy-L-lysine (Fig. 6b).

## Discussion

Reproductive performance is an economically important trait in livestock, which influences the productivity and profitability of farming. The fecundity of goats is mainly influenced by several factors, including litter size, conception rates, and number of live kids. Litter size is one of the most critical traits, as it has a direct influence on profitability of the goat farming. Moreover, small ruminants are renowned for their ability to multiple births in a litter, averaging 1.30–2.37 kids/litter^17^. Although few studies in the recent past were focused on the litter size impact on rumen fermentation, microbiome profiling, and all this association with the performance of offspring^5^. However, this is the first, according to our best knowledge that integrating the information related to litter size association with rumen microbiome and metabolomic profile does. Recent studies indicate that rumen microbiota and metabolites could potentially influence male reproductive performance^18^. Therefore, the overall aim of the present study was to investigate differences in gut microbiota and metabolites of goats that have high or low litter sizes. Rumen microbiota profiling was performed via 16 s rRNA gene sequencing that primarily targets bacterial species, in conjunction with metabolome profiling to characterize the full spectrum of metabolites including nucleic acids, proteins, lipid macromolecules, and other small molecules^19^.

Metabolome profiling indicated that several gut metabolites like methyl linoleate, acetylursolic acid, O-desmethyl venlafaxine glucuronide, melanostatin, and arginyl-Hydroxyproline exhibited significant differences in abundance between the high and low litter size treatment groups. Notably, arginyl-hydroxyproline is a metabolite of Hydroxyproline, which is closely linked to the abundance of Bacteroides, Staphylococcus, and Bifidobacterium^20^, and plays a role in a diverse range of biological functions, including collagen synthesis, blood–brain barrier integrity, and oxidative stress modulation^21^. Taken together, these metabolites may indicate alterations in microflora that in turn may modulate rumen health.

Rumen microbiota is key in rumen development, functioning, metabolism, and regulation. Thus, in this way, it features several phenotypic characteristics including the nature of fermentation products^22^, feed efficiency^23^, production^24^, and reproduction^25^ performance of the ruminants. However, relatively little is known about the influence of litter size on rumen microbiota on female reproductive performance in ruminants. In this study, litter sizes showed a significant impact regarding changes in the structure of the ruminal microbiota suggesting higher litter size has a drastic impact on the rumen microbiome. In this study, 16S rRNA gene-based microbiome profiling indicated that Firmicutes (55.34%) and Bacteroidetes (39.62%), are involved in carbohydrate and protein degradation^26^. These results are consistent with Wang et al.^5^ as they also reported these two bacterial phyla most abundant in the rumen of offspring goats originated from different litter sizes. Moreover, *Firmicutes* and Bacteraioidota are established prominent bacterial phyla of the rumen ecosystem in different ruminant species^27,28^. Previous studies also indicate that the Firmicutes play an important role in the degradation of non-fibrous material^29^, while Bacteroidetes are known to produce several carbohydrate-degrading enzymes encoded by genes that are frequently clustered together in the genome of these organisms, contributing to the breakdown of the structural polysaccharide^30^. At the phylum level, the relative abundance of Proteobacteria appeared to be positively correlated with litter size. Proteobacteria, belonging to a class of protists, can phagocytize bacteria and other organic matter, adapting to diverse gut environments through morphological changes and regulating nutritional metabolism in the gastrointestinal tract^31^. In this study, litter size impacted the ratio of both these bacterial phyla, as in our research their ratio had significant differences. These results are consistent with Wang et al.^5^ as they also reported that goats originating from different litter sizes have changed the ratio of Firmicutes to Bacteraioidota. These phyla are associated with polysaccharide fermentation and thus, in this way allow greater dietary energy availability for host utilization and tissue storage^32^. At the genus level, the relative abundance of Ruminococcaceae_UCG-010, Sediminispirochaeta and Paraprevotella appeared to be positively correlated with litter size, while the relative abundance of Howardella tended to be negatively correlated with litter size, and these results are largely in keeping with similar studies in pigs^33^, where Ruminococcaceae_UCG-010 was found to differ significantly in abundance between sows with different litter sizes and gestation stages. In the gastrointestinal environment, Ruminococcaceae_UCG-010 provides energy for the host by converting plant cellulose and hemicellulose to short-chain fatty acids^34^. A previous study showed that estrogens and estrogen metabolites were associated with the presence and abundance of Ruminococcaceae, which have estrogen-metabolizing properties^35^. Therefore, Prevotellaceae UCG-003 may contribute to higher levels of estrogens, which in turn contributes to increased litter size. Further investigations are needed to investigate this relationship.

## Conclusion

In conclusion, the changes in the abundance of rumen microorganisms, especially Escherichia- Ruminococcaceae_UCG-009, Sediminispirochaeta and Paraprevotella, and the concentrations of metabolites, especially methyl linoleate, acetylursolic acid, O-desmethylvenlafaxine glucuronide, melanostatin, arginyl-Hydroxyproline, might affect the reproductive performance in goat. These findings, encompassing changes in metabolites and shifts in microbial communities, offer valuable insights into the biological basis of fertility traits in goats.

## Data Availability

The datasets presented in this study can be found in online repositories. The names of the repository/repositories and accession number(s) can be found below: NCBI SRA (accession: ID: SUB13987286 Release date: 2024-12-31).
